# Modern treatment strategies in pediatric oncology and hematology

**DOI:** 10.1007/s12672-023-00658-7

**Published:** 2023-06-14

**Authors:** Katarzyna Adamczewska-Wawrzynowicz, Anna Wiącek, Aleksandra Kozłowska, Klaudia Mikosza, Lidia Szefler, Weronika Dudlik, Shreya Dey, Noel Varghese, Katarzyna Derwich

**Affiliations:** 1 Institute of Pediatrics, Department of Pediatric Oncology, Hematology and Transplantology, Poznan University of Medical Sciences, Szpitalna 27/33 street, 61-572 Poznan, Poland; 2 Faculty of Medicine, Poznan University of Medical Sciences, Poznan, Poland

**Keywords:** Pediatric oncology, Neoplasm, Treatment, Targeted therapy, Immunotherapy

## Abstract

Every year, approximately 400 00 children worldwide are diagnosed with cancer. Although treatment results in most types of childhood neoplasms are excellent with survival more than 80%, there are some with poor prognosis. Also recurrent and resistant to treatment childhood cancer remain a therapeutic challenge. Besides chemotherapy, which has been the basis of cancer therapy for years, molecular methods and precisely targeted therapies have recently found their usage. As a result of that, survival has improved and has positively impacted the rate of toxicities associated with chemotherapy (Butler et al. in CA Cancer J Clin 71:315–332, 2021). These achievements have contributed to better quality of patients' lives. Current methods of treatment and ongoing trials give hope for patients with relapses and resistance to conventional chemotherapy. This review focuses on the most recent progress in pediatric oncology treatments and discusses specific therapy methods for particular cancers types of cancer. Targeted therapies and molecular approaches have become more beneficial but research need to be continued in this field. Despite significant breakthroughs in pediatric oncology in the last few years, there is still a need to find new and more specific methods of treatment to increase the survival of children with cancer.

## Introduction

Approximately 400,000 children and adolescents worldwide develop cancer every year [2]. Although the current curability is estimated to be over 80% [1], pediatric cancers outcomes for those who do not respond or relapse is still a significant challenge. Molecular diagnostic advances have resulted in numerous breakthroughs in the diagnosis and treatment of childhood cancer. They have introduced immunotherapy and targeted therapies as the parrarel methods of treatment next to traditional models which are associated with many long-term side effects, especially toxicity. We review advances in therapeutic methods in pediatric hematology and oncology with respect to the most common types of neoplasms in children.

### Treatment of hematologic malignancies

Hematological malignancies usually begin in blood-forming tissues of the body, most commonly the bone marrow, and include leukemias and lymphomas. They account for more than half of all cancer cases in children and are one of the most common cause of death in children aged 1–14. Recent modern treatments have resulted in improvement in survival rates, with approximately 8 in 10 diagnosed patients surviving the disease.

## Acute lymphoblastic leukemia (ALL)

ALL is the most common pediatric cancer, with excellent treatment results and 5 year overall survival (OS) above 90%. Although most children with ALL respond to traditional chemotherapy, there is still a significant minority, including especially patients resistant to treatment and relapsed ALL, in need of new therapies. The knowledge of genetic and moleculardifferences of ALL has led to the development of targeted therapies [3].

### Targeted therapies

Philadelphia chromosome-positive (Ph +) ALL, before the introduction of combined chemotherapy and targeted tyrosine kinase inhibitors (TKIs), was the subgroup of ALL with poor outcome. The first generation of TKIs (Imatinib) markedly exceeded the survival rate of children diagnosed with Ph + ALL. Dasatinib represents the second generation of TKIs. It has inhibiting potency due to its association with non-BCR/ABL kinases. Clinical trials proved the effectiveness and safety of Dasatinib combined with traditional chemotherapy in ALL [4]. Clinical studies of the third generation of TKIs (Ponatinib) have also shown potential, but further tests with accurate dose findings are required [5].

### Immunotherapy

Mortality in relapsed/refractory (R/R) B-cell ALL (B-ALL) is still high. Maintaining a stable remission is still challenging [6]. The development of new immunotherapy led to prolonging OS and disease-free survival (DFS) in pediatric patients with Ph- R/R B-ALL. Blinatumomab, a bispecific antibody that binds CD19 + on B cells and CD3 + on T cells, proved its effectiveness in achieving harmful Minimal Residual Disease (MRD) status, which allows qualification for hematopoietic stem cell transplantation (HSCT) [7, 8]. Cytokine release syndrome and neurological adverse events remain higher in patients treated with Blinatumomab in comparison to traditional chemotherapy [3, 7]. Blinatumomab has approval from the FDA for use in pediatric patients in the treatment of R/R Ph- B-ALL and Ph + B-ALL resistant to TKIs [9].

Inotuzumab Ozogamicin (InO) is monoclonal antibody conjugated to Calicheamicin with CD22 on B cells as a target. InO had a better remission rate in CD22 + R/R B-ALL in adults than conventional chemotherapy. The pediatric clinical trials are focusing on finding a proper dose for children, which would limit the toxicities like post-HSCT sinusoidal obstruction syndrome associated with it [10, 11].

Another immunotherapy treatment is highly specialized chimeric antigen receptor (CAR) T-cells therapy, which involves genetically modifying a patient’s T-cells with fusion proteins that lead them to kill cancer cells, regardless of the patient’s MHC. The main target in ALL is CD19, which is located on B cells, but other targets are also viable [12, 13]. The only CAR T-cell therapy approved by the FDA for use in the treatment of R/R B-cell precursor ALL in pediatric patients is Tisagenlecleucel. The analyses of this treatment estimated the overall remission rate at around 80% [14]. This therapy has revolutionized pediatric oncology and given a chance for long-lasting remission,even with many post-treatment relapses.persistent of memory cells is necessary to ensure the protection needed to avoid potential relapse. It is also known as a bridge to HSCT [13]. Together with the success of the CAR T-cell therapy, new challenges have arisen. One of them is CD19 antigen negative relapse and the absence of CD19 antigen on blast cells due to loss or downregulation [15]. The new potential target—CD22 is already being tested in clinical studies for CD19-negative B-ALL treatment [16]. The main adverse effects of CAR T-cell therapy are similar to Blinatumomab’s—cytokine release syndrome and neurotoxicity.

## Acute myeloid leukemia (AML)

AML is a heterogeneous disease that constitutes about 15–20% of pediatric leukemias. It is a hematopoietic disease presented by clonal expansion of myeloid stem cells. Myelodysplastic syndrome (MDS) frequently predates AML in adults but in pediatric patients, AML commonly appears de novo [17]. This hematological malignancy treatment is remarkably challenging by virtue of poor prognosis (25–30% relapse) and incompletely understood molecular landscape [18]. Therefore we require new modern therapies for pediatric cases.

### FLT3 and KIT inhibitors

In the last few years, there has been a significant improvement in recognizing the genetic basis of childhood AML. Research in the TARGET project [19] has revealed two specific genes that are distinctly mutated in children, AML-KIT and FLT3.

Approximately 30% of patients diagnosed with AML for the first time have mutations in FLT3. These are associated with poor prognosis and lower survival rates. The current first-line therapy for children with an FLT3 mutation is intensive chemotherapy, followed by allo-HSCT. However, it is associated with numerous side effects and high rates of relapse. The first-generation FLT3 inhibitor is Sorafenib which helps treat mutated and wild-type FLT3 genes. Studies show an increased potency of Sorafenib when used in combination with chemotherapy [20]. Other FLT3 inhibitors include Sunitinib, Midostaurin (used in addition to standard chemotherapy), and Lestaurtnib.

For pediatric cases with KIT gene change, the therapy includes the multikinase inhibitor Dasatinib and multi-agent chemotherapy [18].

### Immunotherapy

Immunotherapy treatment of pediatric AML comprises using a patient’s immunology system using CAR T cells. This therapy is an urgent need for young patients due to lack of the long-term side effects associated with traditional chemotherapy. Recent findings from 2021 have exposed the differences between adult and children’s therapy. For instance, CD123 is over-expressed in adult AML but not in pediatric cases. Several studies have shown high expressions of CLEC12A and CD33 in blasts of pediatric patients [21]. Thus, this combination is selected as targets in pediatric AML.

### DOT1L and BRD inhibitors

DOT1L is a methyltransferase involved in the proliferation and differentiation of AML cells and its inhibition is a target for novel AML therapies. For cases of NPM1 mutant AML, using DOT1L inhibitors combined with MLL inhibitors gave positive results [22]. The Bromodomain and Extra-Terminal Domain (BET) family of proteins, both connected to gene transcription regulation, are another recently identified target for therapy. BET inhibitors’ anti-leukemic activity has been analyzed in the treatment of adult AML [18] and serves as potential therapies for pediatric patients as well.

### Menin-MLL interaction

Mutations in the MLL gene appear in 18% of pediatric cases and are common specifically for infants less than a year old. MLL-fusion proteins give rise to the malignant transformation of progenitor cells and stem cells. There are new findings of a novel inhibitor – VTP50469, which suppresses Menin-MLL interactions. This small molecule decreases the activity of several target genes like HOXA, and MEIS1, and reduces neoplasm transformation [23].

## Chronic myeloid leukemia (CML)

CML is a myeloproliferative disease presenting with abnormalities in granulocyte proliferation, massive splenomegaly, and markedly increased white blood cell count. It is associated with the oncogenic BRC-ABL1 gene, created by the translocation of TK ABL on chromosome 9 to the BCR gene on chromosome 22. More than 90% of pediatric patients with the disease have the BCR-ABL tyrosine kinase [24]. CML constitutes about 2–3% of leukemias in pediatric patients under 15 years. The course of the disease in adults is usually milder compared to children and adolescents [25]. The long-term treatment of CML for pediatric patients is analogous to therapy for adults, and it involves tyrosine kinases inhibitors (TKI): first-generation TKI: Imatinib, second-generation: Dasatinib, Nilotinib and third-generation such asBosutinib [26]. However, all clinical evidence indicates that a continued search for unique therapy guidelines adapted for children is required.

### Allogeneic hematopoietic stem cell transplantation (alloHSCT)

CML therapy began with the allogeneic transplant of hematopoietic progenitors and was accompanied by standard chemotherapy (Hydroxyurea) [25]. Allo-HSCT is associated with the risk of graft-versus-host disease (GvHD), mortality related to transplant, and the impossibility of finding a donor. Stem cell transplantation is therefore a third-line treatment for most pediatric CML cases now. Nevertheless, it confers a possibility to cure the disease. However, this procedure of treatment has been gradually replaced by Tyrosine Kinase inhibitors (TKI). They also stop overactive pathways (JAK/STAT, etc.), which block carcinogenesis [26].

### Imatinib

This TKI is the first-line treatment option for pediatric patients with CML in the chronic phase (CP). It disrupts the protein kinases transduction pathways. Imatinib is an oral drug targeted at the BCR-ABL protein. However, this therapy affects children's growth and puberty and is associated with musculoskeletal pain and general malaise. Resistance and intolerance to first-generation TKI has led to the development of a second-generation [27].

### Dasatinib

The second line therapy is Dasatinib (in case of inefficient Imatinib therapy in 25–29% of patients). It inhibits both BRC-ABL and other Tyrosine Kinases like PDGFR and Src [28] Dasatinib can also be used in cases of Nilotinib resistance.

### Nilotinib

Nilotinib is another second-generation TKI intended for pediatric patients with Ph + CML-CP from one year of age or children with CML resistant to Imatinib or Dasatinib.

### Bosutinib

Bosutinib is a third-generation TKI approved for patients, who donnot tolerate Imatinib or have leukemia resistant to first-line TKI.

## Hodgkin’s lymphoma (HD)

Classic Hodgkin Lymphoma (HL) is a lymphoid malignancy derived from B-cells. Several risk factors like EBV infection or mutations in NF-κB pathway genes can cause the development of this disease. One of its features is the presence of large binucleate or multinucleated neoplastic cells or mononuclear variants. They are called Hodgkin Reed-Sternberg (HRS) cells, and they are generally located in lymph nodes. HL occurs to be almost biologically identical in children and adults. What differentiates them is the relative incidence of specific histological subtypes. Classic Hodgkin Lymphoma is divided into four subtypes: lymphocyte-rich CHL, lymphocyte-depleted CHL, mixed cellularity CHL, and nodular sclerosis CHL. HL is the most commonly diagnosed cancer in adolescents (aged 15–19 years) [26]. For children aged 0–14 years, HL represents 4% of all children's cancers. HL has long-term survival rates of more than 90% after treatment with chemotherapy alone or combined with radiotherapy (RT).

### Biomarkers

CD30 and NF-κB have been identified as potential biomarkers in pediatric HL patients, and these molecules may represent therapeutic targets. When CD30 is overexpressed, the activation of transcription factors NF-κB takes place. Physiologically CD30 is mainly expressed on HRS cells and is not present in most healthy human tissues. This makes it an excellent target for directed therapy. Studies have shown that high levels of CD30 + RS cells correlate with poor survival.

Nuclear Factor—κB proteins appear as a family of transcription factors that consists of p65 (RelA), RelB, c-Rel, NF-κB1, and NF-κB2. They are responsible for the expression of genes associated with the immune response, cell proliferation, tumor metastasis, and inflammation. The NF-κB pathway can be triggered by tumor necrosis factor (TNF-alpha). Indicative of HRS cells is the constitutive NF-κB activation caused by NF-κB signaling being dysregulated.

### Treatment

The type of pediatric HL treatment is based on risk group stratification, which depends on factors such as presence of B symptoms, lymph nodes mass, extranodal disease and response to initial treatment, assessed after first weeks of chemotherapy with PET imaging.

When it comes to a treatment strategy for favorable-risk pediatric patients, omission of RT (in early-stage pediatric HL patients) and the use of chemotherapy only is justified. Commonly used medication are Vincristine, Etoposide, Prednisone, and Doxorubicin.

Even though there has been remarkable progress in the treatment of pediatric HL, there is still a significant number of cases (10–25% of patients) of relapsed/refractory (R/R). Treatment is based on salvage chemotherapy, high dose chemotherapy (HDCT), followed by autologous stem cell transplantation (autoSCT). Treating R/R patients is a therapeutic challenge because of low survival (ranging from 18 to 41%).

### Targeted therapy

Targeted therapy of pediatric Hodgkin lymphoma comprises of monoclonal antibodies, signal transduction inhibitors, immunotherapy, and epigenetic agents (histone deacetylase (HDAC) inhibitors). A commonly used anti-CD30 antibody–drug conjugate, involved in inducing apoptosis of HRS cells, is Brentuximab vedotin (Bv), which currently comprises, alongside with chemotherapy, first line treatment in advanced stage HD. Another monoclonal antibody 5r4tzis Nivolumab. Its role is to block the PD-1 receptor expressed on peritumoral T cells. Apart from these monoclonal antibodies, Bortezomib is also used in HL therapy. It selectively inhibits the 26S proteasome stabilizing the NF-κB inhibitor, which is normally degraded by the ubiquitin–proteasome system. Treatment of pediatric HL may also include the use of Vorinostat and Panobinostat. These are the histone deacetylase inhibitors that are involved in cell cycle arrest and apoptosis. Vorinostat also decreases the amount of Th2 cytokines and chemokines like TARC responsible for protecting tumor cells from the immune system. Moreover, HDAC inhibitors have been shown to interact synergistically with proteasome inhibitors to induce apoptosis. These findings might contribute to remarkable clinical outcomes in the future [29].

## Non-hodgkin’s lymphoma (NHL)

Non-Hodgkin lymphoma (NHL) is a type of cancer that develops from lymphocytes. Mature B-cell non-Hodgkin lymphomas (B-NHLs) constitute approximately 60% of all NHL diagnoses [30]. The most common pediatric B-NHLs are Burkitt lymphoma, diffuse large B-cell lymphoma (DLBCL), and primary mediastinal B-cell lymphoma (PMBCL). Treatment for children with PMBCL is generally superior to that observed in adults [31]. On the other hand, therapy for DLBCL and Burkitt lymphoma, due to high doses of methotrexate, cyclophosphamide and anthracyclines, as well as irradiation has a high incidence of both short and long-term toxicities.

### Biomarkers

Burkitt lymphoma is a germinal center mature B-NHL. It is associated with the expression of CD10, CD19, CD20, and CD22. In most cases, the proliferation marker Ki67 is expressed and MYC oncogene. MYC transcription factor plays an essential role in tumor pathogenesis and development.

DLBCL shows a lower expression of the proliferation marker Ki67. Two-thirds express the anti-apoptosis protein Bcl-2 and one-third show the MYC transcription factor. When these two proteins are co-expressed, which happens in 10% of germinal center DLBCL cases, the prognosis worsens. It is associated with the expression of light chain-restricted surface IgG and B-cell markers CD19, CD20, CD22, CD79a, and PAX-5.

### Treatment

Currently, NHL treatment is based on chemotherapy with the addition of immunotherapy. It has become the standard of care for most intermediate and high-risk pediatric mature B-NHL. When it comes to pediatrics, both DLBCL and BL are treated similarly. Usually, Rituximab is incorporated into frontline therapy. It’s a human monoclonal antibody with a high affinity against CD20 on B cells. Other than for a small proportion of patients with localized disease, the treatment emphasizes the importance of central nervous system–directed therapy, including high-dose Methotrexate. The outcomes with these approaches have been excellent with EFS and overall survival (OS) is > 90% in early-stage patients and > 80% in patients with advanced-stage disease [32]. The outcomes of treating PBMCL are not as excellent as those associated with DLBCL or BL. In an attempt to improve the outcome, the DA-EPOCH-R (Etoposide, Prednisone, Vincristine, Cyclophosphamide, Doxorubicin, and Rituximab) regimen was conducted. Early analysis of the study (47 patients accrued) at a median follow-up of 27 months demonstrated an EFS and OS of 72% and 82% [33].

#### Treatment of solid tumors in children

Although the majority of pediatric malignancies are hematologic, solid tumors represent approximately 30% of them. Cancer of the brain particularly is prevalent, with it being 26% of all cancers in children under 15 [34]. Other common types include Neuroblastoma (15%), Rhabdomyosarcoma (7%), Wilms tumor (6%), Ewing sarcoma (8%), Retinoblastoma (5%), and other miscellaneous tumors. However, treatment of these has improved considerably in the past few decades due to research and a better understanding of their molecular and cellular basis. Most cure rates have improved by more than 50% than in the 1970s [35].

## Neuroblastoma (NB)

Neuroblastoma is one of the most common solid cancer in pediatric patients and accounts for 6–7% of all neoplasms in children aged 0–14, and is the most common in children under the age of one [36]. It is an embryonal tumor of the sympathetic nervous system, is derived from neural crest cells, frequently localised in the adrenal gland. In recent years, the prognosis of patients with high-risk Neuroblastoma has significantly improved due to the use of immunotherapy. Neuroblastoma cells (besides the tumor stage) have a high level of expression of GD2 ganglioside, which is located on the outer cell membrane. Dinutuximab is the first approved anti-GD2 monoclonal antibody and its introduction into treatment has greatly improved outcomes. MoAbs bind to the tumor and engage granulocytes and NK cells, which kill tumor cells through their cytotoxicity. In addition, MoAbs also induce complement-dependent cytotoxicity, which results in the formation of membrane attack complexes and cell lysis. The therapeutic process is positively influenced by the fact that the level of GD2 circulating in the blood and the cerebrospinal fluid is relatively low and does not interfere with the binding of the medication to cellular GD2 [37].

Amplification of the MYCN gene has been identified in nearly half of all high-risk cases of Neuroblastoma. They are key regulators of cell survival and growth. The Bromodomain and Extra Terminal (BET) family of proteins regulate the expression of the MYCN genes. Recent research has shown that selective inhibition of the BET proteins led to potent downregulation of the MYCN genes in young patients with Neuroblastoma as well as various other solid malignancies [38]. Furthermore, BET inhibitors have also been shown to silence Bcl-2 proteins leading to cytotoxicity [39]. Therefore, this could be a potential therapeutic target for the future.

## Brain tumors

Tumors of the brain and CNS are the most common cancer-related cause of death in children. The most frequently occurring are pediatric low-grade gliomas (pLGG). Although surgical management of brain tumors is the treatment of choice, it is not always possible for deeper-seated or infiltrative tumors. Chemotherapy and radiotherapy used in this case are associated with many long-term adverse effects. The recognition of the molecular basis of pLGG demonstrated that it is in large part connected with the upregulation of the RAS-mitogen-activated (RAS/MAPK) pathway, which created opportunities for using inhibitors of this pathway as a treatment [40]. Pediatric high-grade gliomas are a heterogeneous group of brain tumors, and as there is no universal therapy, there are clinical trials of targeted therapies referred to individual subgroups.

Nevertheless, none of the new therapies successfully extended the OS, which remains extremely low [41]. The most common malignant pediatric brain tumor is medulloblastoma (MB) accounting for almost 20% of all childhood CNS malignancies. The standard treatment contains surgical intervention, radiotherapy, and chemotherapy but still is associated with high morbidity. The main point of interest in clinical studies is targeted therapy based on hedgehog pathway inhibitors, which are already approved as a treatment for different types of cancer. However, their use in MB is still in the phase of clinical trials [42]. Bevacizumab shows promise due to the high expression of VEGF but has had variable responses [43]. Inhibition of MYCN genes as previously discussed also holds potential in cases of pediatric Neuroblastoma. Lastly, autologous stem cell rescue preceded by high-dose chemotherapy has shown to improve survival rates in certain cases, albeit with a high ratio of morbidity [44]

## Osteosarcoma

Osteosarcoma (OS) is the most common malignant bone tumor [45]. It occurs most commonly in young people and affects more males than females. The most common subtype of osteosarcoma is conventional central Osteosarcoma [46]. The overall 5 year survival rate for children ages 0–18 with Osteosarcoma is 68% [47]. The best treatment for children’s oncology group is the combination: Cisplatin, Doxorubicin, and high-dose Methotrexate [47]. Most patients are diagnosed with localized tumors. The effects of conventional chemotherapy in locoregional Osteosarcoma are rewarding and still used in these cases. Other additional and experimental treatments in non-local or progressive disease consist of “targeting the bone microenvironment (Bisfosfonates), tyrosine kinase receptor (e.g., Sorafenib, Pazopanib), and intracellular signaling molecules (Dasatinib)” [48].

Mifamurtide (Mepact) is a synthetic lipophilic analog of muramyl dipeptide, tested as a drug in immunotherapy in case of localized disease. This immunostimulant is used as an additional and complementary drug to conventional chemotherapy [47, 48]. Research has shown that the risk of progression was 5 times lower for the Mifamurtide group [48]. To confirm Mifamurtide’s impact on survival benefits, researchers need more analyses on bigger groups of examined patients, which might be done in the near future. There is currently no immunotherapy that has been clearly demonstrated to be effective against OS [45].

Cabozantinib, MET/VEGRF2 inhibitor, has shown promising results in unresectable and relapsed Osteosarcoma and Ewing sarcoma. Phase II of CABONE research (NCT02243605), which included patients only with confirmed progressive disease, demonstrated the activity of Cabozantinib in advanced and recurrent disease but warrants further investigation [49].

## Ewing sarcoma

Ewing sarcoma is a malignant tumor that usually grows in bone. It is most common in children and adolescents between ages 10 and 19 [50]. Moreover, it can also grow in soft tissues which are connected to the diseased bone, which most often are long bones such as the femur, tibia, humerus, and pelvis [51]. The 5 year relative survival rate for Ewing sarcoma is on average 61% [52]. However, patients with metastases have much lower rates, and therapies carry numerous short- and long-term side effects.

In an ongoing clinical trial (NCT02657005), patients with metastatic, relapsed Ewing’s sarcoma are administered the standard chemotherapy drug—Vincristine – combined with TK216, which is an inhibitor of ETS proteins [53] Another trial has also studied the addition Talazoparib or Niraparib (selective PARP inhibitors) to Irinotecan and Temozolomide. Early results showed potential at lower doses [54].

## Rhabdomyosarcoma (RMS)

**Fig. 1:**
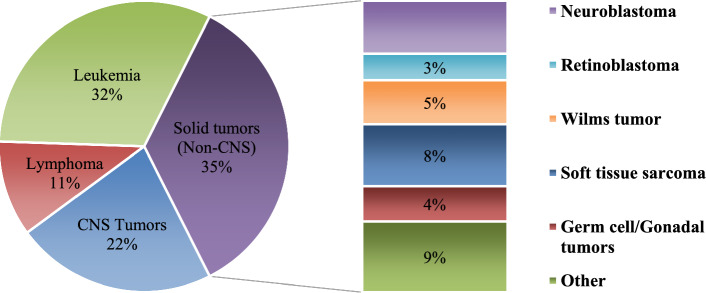
Distribution (%) of pediatric cancers (0–14 years)

**Fig. 2:**
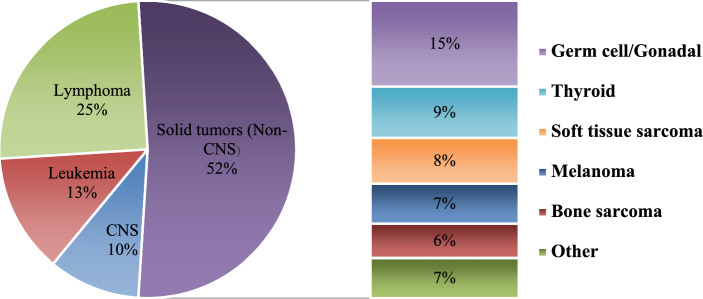
Distribution (%) of pediatric cancers (15–19 years)

Rhabdomyosarcoma accounts for 3% of all diagnosed cancers in the group of pediatric patients. There are two subtypes of RMS: embryonal RMS (ERMS), which is the most common, and alveolar RMS (ARMS) [41]. The prognosis of ARMS is much worse, and it is more clinically aggressive than ERMS. Today, low- and medium–risk patients without metastases treated with frontline multi-modality therapy have excellent outcomes. However, high-risk patients or patients who have experienced metastases and disease relapses are a serious therapeutic challenge Figs. 1, 2.

In low-risk patients, the focus has been on lowering Cyclophosphamide doses and reducing treatment-related toxicity (myelosuppression, infectious complications, and infertility) with no reduction in treatment efficacy at lower doses [55]. The use of maintenance chemotherapy offers hope for improvement in managing patients with high-risk RMS. The drugs administered were Vinorelbine with a low dose of Cyclophosphamide [56]. Vinorelbine inhibits microtubule polymerization and therefore has a cytostatic effect [46]. Open-label, randomized, phase III clinical trials (showed a significant improvement in 5 year overall survival in patients who received maintenance chemotherapy [56, 57]. The obtained results directed research to add Bevacizumab (monoclonal antibody) and Temsirolimus (selective protein kinase inhibitor) to Vinorelbine’s treatment. However, the results of these studies are not available yet [58].

Phosphodiesterase type 5 (PDE5) overexpression has also been observed in various malignant tumors including Rhabdomyosarcomas. A recent study has shown that Sildenafil (PDE5 inhibitor), when combined with chemotherapeutic drugs like Doxorubicin, have increased their cytotoxic effects while potentially reducing side effects. Mechanisms involved in this process are increased cell apoptosis and ROS production [59].

## Wilms tumor

Wilms tumor is the most common renal cancer in the pediatric age group [60], generally with a higher incidence in girls. Wilms tumor is usually diagnosed between 3 and 4 years old [49]. Currently, the 5 year survival rate of children with Wilms tumor is about 90%. Therefore targeted therapy and immunotherapy are rarely used in Wilms tumor treatment. The sites in targeted therapy research are mainly focused on the Insulin-like growth factor 2 (IGF2) pathway, anti-angiogenesis (especially concerning the VEGF pathway), PI3K signaling pathway, and some miRNAs as a targeted drug [60].

IGF1R, the IGF2 receptor, is currently considered the most feasible therapeutic target due to its overexpression and major role in the occurrence and growth of cancer. Furthermore, research has shown a correlation between a higher IGF1R copy number and a shorter relapsed–free survival time [51, 60].

Wilms tumor immunotherapy consists of three limbs: inhibition of the COX-2 pathway (to inhibit tumor immune escape), chimeric antigen receptor (CAR)-T cell therapy (Glypican-3, EGFR, and B7-H3), and multi–tumor associated antigen (TAA)- specific cytotoxic T lymphocytes (CTL) therapy. The phase I clinical trial (f multi-TAA-specific CTL therapy has been completed and gave satisfactory results [60].

## Conclusion

**Fig. 3:**
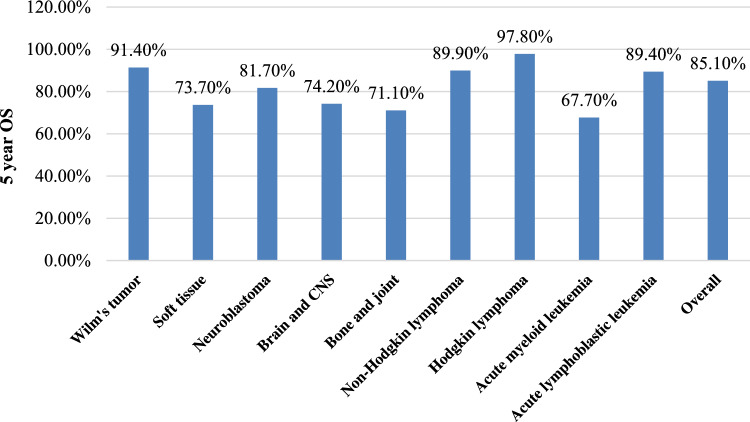
Five year overall survival rates of pediatric cancers (0–19 years)

Although a significant breakthrough in treating pediatric oncologic patients has occurred in the last few years (Fig. 3), leading to an increase in overall survival rates of almost all the various pediatric malignancies, there is still the need to find new and more specific methods of treatment to increase the survival of children with cancer.

Targeted therapies and molecular approaches have become beneficial for young patients due to lower toxicity and long-term side effects, but research must continue in this field.

## Data Availability

Not applicable.
